# Interpretations of the Role of Plasma Albumin in Prognostic Indices: A Literature Review

**DOI:** 10.3390/jcm12196132

**Published:** 2023-09-22

**Authors:** Kim Oren Gradel

**Affiliations:** 1 Center for Clinical Epidemiology, Odense University Hospital, 5000 Odense, Denmark; kim.gradel@rsyd.dk; Tel.: +45-21-15-80-85; 2 Research Unit of Clinical Epidemiology, Department of Clinical Research, University of Southern Denmark, 5000 Odense, Denmark

**Keywords:** plasma albumin level, role of inflammation, role of nutrition, prognostic indices

## Abstract

This review assesses how publications interpret factors that influence the serum or plasma albumin (PA) level in prognostic indices, focusing on inflammation and nutrition. On PubMed, a search for “albumin AND prognosis” yielded 23,919 results. From these records, prognostic indices were retrieved, and their names were used as search strings on PubMed. Indices found in 10 or more original research articles were included. The same search strings, restricted to “Review” or “Systematic review”, retrieved yielded on the indices. The data comprised the 10 latest original research articles and up to 10 of the latest reviews. Thirty indices had 294 original research articles (6 covering two indices) and 131 reviews, most of which were from recent years. A total of 106 articles related the PA level to inflammation, and 136 related the PA level to nutrition. For the reviews, the equivalent numbers were 54 and 65. In conclusion, more publications mention the PA level as a marker of nutrition rather than inflammation. This is in contrast to several general reviews on albumin and nutritional guidelines, which state that the PA level is a marker of inflammation but not nutrition. Hypoalbuminemia should prompt clinicians to focus on the inflammatory aspects in their patients.

## 1. Introduction

Prognostic index models can be used either for prediction or explanation [1]. A prediction model aims to generate a risk score that grades patient prognosis. However, the main limitation of prediction models is that they do not explain the impact or role of the model’s individual co-factors. As an example, the APACHE III prognostic index [2] often yields high performance, but it does not account for the impact or role of each of its numerous co-factors. Such assessments demand explanatory models which aim to advance scientific knowledge by interpreting the role of individual co-factors [1].

Hypoalbuminemia is consistently associated with adverse outcomes, and it is considered a strong prognostic predictor [3,4,5].

In contrast, the possible pathological mechanisms behind hypoalbuminemia are more obscure, and they have been debated for decades [6,7,8,9]. For the vast majority of diseases (such as malignancies, diabetes, or sepsis), reasons for hypoalbuminemia are difficult to assess in observational studies because the level of serum or plasma albumin (PA) depends on both synthesis, breakdown, external loss, and the distribution between the vascular and extra-vascular space [10]. 

For some diseases related to specific organs (liver or kidneys), hypoalbuminemia can partly be related directly to these disorders (e.g., reduced synthesis in patients with cirrhosis [11]). Among the mechanisms not directly related to specific organs, two main explanations have dominated the reasons for hypoalbuminemia: malnutrition and inflammation [8,10,12]. As malnutrition and inflammation are positively correlated in many diseases, hypoalbuminemia is often statistically associated with both of these.

This literature review provides an overview of all retrieved prognostic indices that incorporate PA. For indices that appear in at least 10 prognostic studies, we will describe how PA is interpreted based on the 10 newest research articles and reviews. 

## 2. Materials and Methods

### 2.1. Initial Literature Searches

To obtain an overview of publications that included prognostic indices with PA levels, we used the search terms “albumin AND prognosis” in the PubMed database (https://pubmed.ncbi.nlm.nih.gov/) without any restrictions. A search on 22 December 2017 yielded 18,007 results, for which all titles and abstracts were examined to see whether the publications comprised such indices. This search was performed once again on 1 October 2021, yielding 5912 more results. From these initial literature searches, we found the indices shown in Table A1 and Table A2, Appendix A. 

### 2.2. Literature Searches for the Specific Prognostic Indices

Table A1 gives an overview of the search terms used for all the initially retrieved indices as well as the number of hits on the search date (18 February 2022). After automatically retrieving the records, we sorted them chronologically (according to “Most recent”, i.e., not according to “Publication date”) and manually examined the abstracts (newest first) to deduce whether the publications were in agreement with the search terms. In this manual process, we only included English-language publications presenting human studies and excluded casuistic studies. For indices with <10 records, we counted all manually retrieved records, whereas we finished counting at 10 for indices with ≥10 records.

### 2.3. Final Inclusion of Prognostic Indices and Publications

In the final stage, we only proceeded with prognostic indices that had at least 10 manually retrieved publications (Table A1, right column). For these indices, we assessed the 10 newest original research publications as well as a maximum of the 10 newest reviews regarding the indices. The reviews were retrieved using the same search terms as depicted in Table A1 but with restrictions to “Review” and “Systematic review” in the PubMed database. Reviews discussing specific diseases were retrieved on 18 February 2022, whereas reviews regarding broader patient groups were retrieved in week 10, 2023. We only included reviews in which the index was the main topic, regardless of whether the index was evaluated for one or several diseases or patient groups. Thus, we excluded reviews where the main topic was a disease assessed via several prognostic indices. 

### 2.4. Reporting of Results

For each prognostic index, we looked through the 10 newest original research publications and retrieved reviews (where possible) to deduce whether they interpreted the PA-level results. Concerning the research publications, we focused on the abstract, introduction, and discussion sections as these were most likely to contain information about the role of PA. For reviews, we scrolled through all of the text. For all publications, we finalized each search by retrieving all “alb” (case letter insensitive) in the pdf file to deduce whether we had missed sentences that possibly dealt with albumin. If a publication interpreted the PA-level results, we further evaluated whether they reported these as markers of inflammation, nutrition, or other conditions (e.g., liver disorders or external loss). We differentiated between prognostic indices developed for specific diseases and prognostic indices developed for broader patient groups.

We further categorized author affiliations as either two or more countries or a single country (regardless of patient populations). For the latter, we recorded the authors’ continent and country affiliation.

## 3. Results

### 3.1. General Overview

From the initially retrieved 23,919 records, going back to 1957, we found the prognostic indices shown in Table A1. Using the search strings shown in Table A1, we found 38 indices with 10 or more publications in PubMed. In the manual assessments of the PubMed-derived records, we found that 30 of these indices had 10 or more original research studies in English that were not casuistic. For the remaining eight indices, alternative search strings (not shown) were used, but these resulted in either a very low (0–2) or very high number of records. As an example, the search string “serum creatinine to albumin ratio” yielded 3554 records, none of which had the search string per se (PubMed: Quoted phrase not found: “serum creatinine to albumin ratio”), and our manual assessment did not yield relevant studies, as many assessed creatinine and albumin in urine but not in serum or plasma.

Table A2 gives an overview of the formulas used for computing all of the prognostic indices retrieved from our initial assessment of the initially retrieved 23,919 records. These formulas are mainly relevant in the context of inflammation vs. nutrition. C-reactive protein or Body Mass Index (BMI) are examples of other index parameters that are markers of inflammation and nutrition, respectively.

### 3.2. Prognostic Indices That Were Further Assessed

Table 1 gives an overview of the 30 relevant prognostic indices and the diseases or patient groups for which they have mainly been used.

We characterized 12 of these indices as covering specific disorders and 18 as covering broader patient groups. 

### 3.3. Prognostic Indices Mainly Related to Specific Disorders

These 12 indices could further be grouped into the following: lung cancer (ALI), liver disorders (ALBI-T Score, ALBI, Child–Pugh Score, NAFLD Fibrosis Score [NFS]), kidney disorders (Protein Energy Wasting [PEW] Index, Malnutrition–Inflammation Score), specific hematological malignancies (International Prognostic Score for Advanced Hodgkin’s Disease, International Staging System [ISS] for Multiple Myeloma), gastrointestinal bleeding (AIMS65), pneumonia (SMART-COP), or spinal metastases (New England Spinal Metastasis Score) (Table 1). 

Most of the 177 retrieved references were recent; 161 (91.0%) were from 2017 or later, and the oldest reference was from 2009 (Table 2).

### 3.4. Prognostic Indices Mainly Related to Specific Disorders: Original Research Studies

The 12 indices were covered by 119 studies, as one study dealt with both the Child–Pugh Score and ALBI [55] (Table 2). A total of 83 studies (69.7%) did not mention anything about the role of PA. Eight of the nine studies that interpreted PA levels according to “miscellaneous” (i.e., neither due to inflammation nor nutritional status) dealt with prognostic indices specifically generated for liver patients (Table 2) and all related hypoalbuminemia to liver disorders. Among the studies that interpreted PA levels in relation to inflammation and/or nutritional status, 3 studies interpreted PA levels according to inflammation only, 19 studies interpreted PA levels according to nutrition only, and 10 interpreted PA levels according to both inflammation and nutrition.

### 3.5. Prognostic Indices Mainly Related to Specific Disorders: Reviews

For 2 indices, we did not find any reviews, whereas we found from 1 to 10 reviews for the other 10 prognostic indices, meaning that we retrieved 58 reviews altogether (Table 2). Forty-three reviews (74.1%) did not deal with the role of PA in the indices. Among the remaining 15 reviews, 8 related PA to inflammation, 5 related PA to nutritional status, and 10 related PA to miscellaneous factors. Liver disorders were listed as reasons for hypoalbuminemia dominating among the miscellaneous factors [33,62,64,65,105,141,163,164], with two liver disorder indices, ALBI and NFS, showing the greatest level of prevalence. The reasons for hypoalbuminemia among the miscellaneous factors (other than liver disorders) were acidosis [105], PA loss in dialysis [136], and nephrosis [141]. 

### 3.6. Prognostic Indices Mainly Related to Broader Patient Groups: Original Research Studies

Our sample of original research studies included 175 studies with 18 indices related to broader patient groups (Table 1). Five studies discussed 2 of the 18 indices [190,191,192,193,194] (Table 3). 

Nearly all studies were from 2022 or 2021, and the three oldest were from 2016 [373,374,376]. 

Thirty-six studies (20.6%) did not mention any factors that may have an impact on the PA level, including all 10 studies with the APACHE III Score, 7 with the Gustave Roussy Immune (GRIM) Score, 6 with the Platelet–Albumin–Bilirubin (PALBI) Grade, and 4 with the Glasgow Prognostic Score (Table 3). Moreover, 13 studies (including all 10 studies dealing with the Ischemia-modified Albumin/Albumin ratio) only mentioned factors that were neither related to inflammation nor nutrition (e.g., liver disorders). Among the remaining 126 studies, 24 (19.1%) associated the PA level with inflammation only, 37 (29.4%) associated the PA level with nutrition only, and 65 (51.6%) associated the PA level with both inflammation and nutrition. 

Many indices were primarily used for disease entities with a common pathophysiology (Table 4). 

Among the 175 publications, 91 (52%) discussed malignancies, 19 (10.9%) discussed infections, and the remaining 65 (37.1%) included discussions of various other disease entities and patient groups. All 10 studies with the Gustave Roussy Immune (GRIM) Score and the Systemic Inflammation Score dealt with malignancies, which was also the case for 9 of the 10 studies regarding the Albumin/Alkaline Phosphate Ratio; the Glasgow Prognostic Score; the Hemoglobin, Albumin, Lymphocyte, Platelet Score; and the Naples Prognostic Score. 

### 3.7. Prognostic Indices Mainly Related to Broader Patient Groups: Reviews

The 73 retrieved reviews featured 13 of the 18 indices, of which 5 (C-reactive protein/Albumin ratio, COntrolling NUTritional status (CONUT) Score, Geriatric Nutritional Risk Index, Glasgow Prognostic Score, and Prognostic Nutritional Index) had at least 10 reviews (Table 3). Sixty-eight reviews (93.2%) were from 2019 or later. The four oldest reviews (from 1991, 2000, 2008, and 2014) all dealt with the APACHE III Score. 

Six reviews (8.2%) stated that inflammation only had an impact on the PA level, 20 (27.4%) only mentioned nutrition, and 40 (54.8%) mentioned both inflammation and nutrition (Table 3). Factors other than inflammation or nutrition (e.g., liver disorders) were mentioned in 13 reviews (17.8%), whereas 7 reviews (all the 5 on APACHE III and 2 on Glasgow Prognostic Score) did not mention any factors that could have an impact on the PA level. 

Fifty-six reviews (76.7%) dealt with malignancies, five (6.8%) dealt with infections, and the remaining twelve (16.4%) dealt with other diseases or patient groups (Table 4).

### 3.8. Authors’ Continent and Country Affiliations

Three countries (the USA, China, and Japan) accounted for 46.2% of the 119 original research studies mainly related to specific disorders, and 15 studies (12.5%) had authors from two or more countries (Table 5). 

Among the 58 reviews, 14 (24.1%) had authors from two or more countries, 12 (20.7%) were from the USA, 7 (12.1%) were from China, and 6 (10.3%) were from the UK; the remaining 19 reviews had authors from 13 different countries. 

Among the original research studies mainly related to broader patient groups, all except eight had all of their authors affiliated with one country only (Table 5). Among these 167 studies, 148 (88.6%) had author affiliations in Asian countries, of which China, Japan, and Turkey dominated with 72, 40, and 27 studies, respectively. A total of 52 reviews (71.2%) were from authors with affiliations with institutions in China; Taiwan had six reviews (8.2%). Four reviews were from authors from more than one country, and the remaining eleven reviews were from eight different countries. 

## 4. Discussion

Among the 425 original research articles and reviews that included discussions on indices and PA levels, 156 (36.7%) related PA levels to inflammation, and 196 (46.1%) related them to nutrition. For the 248 articles/reviews that discussed broader patient groups, the equivalent metrics were 135 (54.4%) for inflammation and 162 (65.3%) for nutrition. Some manuscripts discussed indices mainly related to specific diseases (e.g., the Child–Pugh Score specifically designated for liver disorders), thus placing less emphasis on inflammation and nutrition.

**Figure 1:**
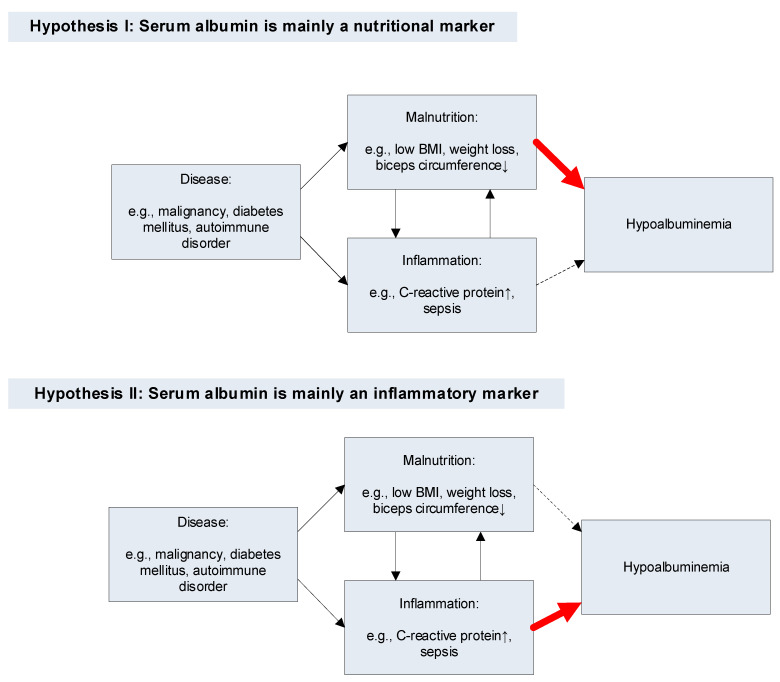
Pathways for hypoalbuminemia according to two hypotheses. Thin black arrow: statistical association. Thick red arrow: main cause of hypoalbuminemia. Thin dashed arrow: weak causal association between the factor (inflammation [Hypothesis I] or malnutrition [Hypothesis II]) and hypoalbuminemia.

Many patient groups are characterized by both inflammation and malnutrition, e.g., in patients with malignancies [437]. Because hypoalbuminemia also often characterizes such patient groups [4], it is statistically correlated with both inflammation and malnutrition. Two models, one in which PA is mainly a marker of nutrition and one in which it is mainly an inflammatory marker, are depicted in Figure 1.

However, PA as a nutritional marker was described as a “myth” already more than three decades ago [438,439]. Since then, many reviews have emphasized PA as being a negative acute-phase protein and, as a result, a marker of inflammation [5,7,8,9,10,440,441,442,443]. Moreover, nutrition guidelines from the USA and Europe have stated that the PA level is not a valid marker of nutritional status [444,445,446]. Perhaps the most convincing studies are those of PA levels in undernourished patients who have no concomitant inflammation. A review comprising 63 studies of such patients, most with anorexia nervosa, found that their PA levels were normal [447]. In one study, 16 anorectic patients had a mean PA level of 40.5 g/L, and 16 healthy controls had 41.1 g/L, both of which were within normal limits (35–50 g/L) [448]. Curiously, another five anorectic patients who were dying had much lower mean PA levels (33.5 g/L); however, these were not ascribed to their nutritional status but to organ failure with concomitant extravasation of the PA. Mechanisms related to factors that determine the PA level are beyond the scope of this review, but we would like to refer interested readers to two excellent reviews that give useful details [6,7]. 

The compelling amount of evidence against the notion of the PA level being a marker of nutrition in conjunction with the influx of recent articles that still describe the PA level as a marker of nutrition prompted us to investigate this systematically. To delineate the topic, indices with PA reported in at least 10 original publications were selected. 

There were 289 studies and reviews (68.0%) from Asia, most of which were from China (160/289 [55.4%]), followed by Japan (59/289 [20.4%]) and Turkey (33/289 [11.4%]). There were no significant differences between continents or countries vs. factors related to the PA level, although the numbers for most of the continents or countries shown in Table 5 are too small to generate useful statistics. The reasons for why so many studies and reviews dealing with the reported indices emanate from Asia are unknown. The authors of all of the general reviews on albumin mentioned above were either from the USA [5,9,440,442,443], Europe [8,10,441], or both [7]. Still, we cannot conclude why so many studies and reviews mentioned hypoalbuminemia as a marker of nutrition. 

The PA level is known to be a strong prognostic predictor regardless of patient population or disease entity [3,4,5], and this is probably well-known by most clinicians. However, according to the many studies and reviews reported here, of which most are very recent, it seems that fewer clinicians are aware of the fact that hypoalbuminemia is not very valid as a marker of nutrition. Specifically, we suspect that many protein drinks, which are not cheap, are given to patients solely due to hypoalbuminemia. Whether this is beneficial for individual patients should be assessed clinically by using factors other than the PA level. The reviews we studied generally related the beneficial aspects of albumin supplementation to factors other than nutrition, such as immunomodulatory, anti-inflammatory, or antioxidant properties [449]; however, whether albumin supplementation is beneficial at all (e.g., for sepsis patients) is also hotly debated [450]. Regardless of this, it is important to focus on inflammatory aspects in patients with hypoalbuminemia. Even in patients with specific organ dysfunctions, such as in the kidney [105,451] or liver [452], inflammation is often an important aspect of pathogenesis and the PA level.

This review has some limitations that need to be addressed. Firstly, this review was written by only one author, who is biased due to his studies that also indicate an important role for inflammation in relation to the PA level [453,454,455,456,457,458,459,460], of which only one study includes nutritional data (the Body Mass Index) [459]. Moreover, this also implies that there was no crosschecking of the scrutinized publications. However, if factors were overlooked or misinterpreted in a few of the references, this probably did not change the overall results. Secondly, the distinction between indices used for specific vs. broader patient groups is partly arbitrary. This distinction was chosen because indices covering broader patient groups are probably more prone to various interpretations than indices that specifically monitor target organs such as the liver. However, indices for specific diseases have also been used for other patient groups, e.g., the Advanced Lung Cancer Inflammation Index (ALI) where seven of the 10 original articles in this review dealt with other malignancies or coronary diseases. Likewise, many of the general indices have mainly assessed certain diseases, e.g., the Naples Prognostic Score where nine of the 10 original studies comprised malignancies. Regardless of these uncertainties, a much higher rate of studies of specific disorders did not discuss reasons for the PA levels (68.9% vs. 20.6% in studies of broader patient groups). Finally, it took some time to look through the PubMed data, which were initially analyzed by skimming 23,919 titles and abstracts to find relevant indices with PA, after which the 425 selected publications were scrutinized. During this process, more publications with the actual indices appeared daily in PubMed. As an example, the search string “advanced lung cancer inflammation index” yielded 54 results on 18 February 2022 (Table A1), but at the time of writing (9 June 2023), the same search string yields 90 results. However, except for reviews of the APACHE III Score, the vast majority of this review’s publications were not more than four years old (Table 1 and Table 2). The studies and reviews published after the ones reported here would probably have little impact on our results, as there is no reason to believe a recent revolutionary change has occurred for a concept discussed for more than three decades.

## 5. Conclusions

Although numerous reviews and nutritional guidelines have advocated against using the PA level as a marker of nutrition, this is still how it is interpreted in many studies and reviews of indices commonly used with albumin. Many of these publications also interpreted the PA level as a marker of inflammation, whereas very few reported it as a marker of inflammation but not nutrition. This may have an impact on the prognosis of patients with hypoalbuminemia if clinicians focus more on nutritional than inflammatory aspects, both of which are important to assess in frail patients. Regarding nutrition, many measures have been proposed, such as body mass index, waist/hip ratio, or body fat content. There is no universally accepted gold standard for assessing a patient’s nutritional status, but the PA level should not be used, solely or partly, to fill this void. In contrast, the PA level is important in assessing the inflammatory status of a patient.

## Figures and Tables

**Table 1:** Prognostic indices with at least 10 original research articles and the diseases or patient groups noted in published studies and reviews.

Index Name (Sorted Alphabetically)	Disease(s)/Patient Group(s)
Advanced Lung Cancer Inflammation Index (ALI)	Various, mainly malignancies
AIMS65	Gastrointestinal bleeding
ALBI-TNM = ALBI-T Score	Hepatocellular carcinoma
Albumin–Bilirubin (ALBI) Score	Liver diseases
Albumin/Alkaline Phosphate Ratio (AAPR)	Various, mainly malignancies
Albumin/Fibrinogen Ratio (AFR)	Various
Albumin/globulin ratio	Solid malignancies
Acute Physiology, Age, Chronic Health Evaluation (APACHE) III Score	Intensive care unit patients
Blood Urea Nitrogen/Albumin Ratio	Various
C-reactive protein/Albumin ratio	Various
Child–Pugh Score	Chronic liver diseases, especially cirrhosis
COntrolling NUTritional status (CONUT) Score	Various, mainly malignancies
Fibrinogen/Albumin ratio (FAR)	Various
Geriatric Nutritional Risk Index	Various
Glasgow Prognostic Score	Malignancies, mainly solid
Gustave Roussy Immune (GRIM) Score	Malignancies
Hemoglobin, Albumin, Lymphocyte, Platelet (HALP) Score	Various, mainly malignancies
International Prognostic score for advanced Hodgkin’s disease	Hodgkin’s disease
The ISRNM PEW index	Chronic kidney disease
International Staging System (ISS) for multiple myeloma	Multiple myeloma
Ischemia-modified Albumin/Albumin ratio	Various
Malnutrition–Inflammation Score = Kalantar Score	Kidney diseases
NAFLD Fibrosis Score (NFS)	Nonalcoholic fatty liver disease (NAFLD)
Naples Prognostic Score	Various, mainly malignancies
Neutrophil (Percentage)/Albumin Ratio (NPAR)	Various
New England Spinal Metastasis Score	Spinal metastases
Platelet–Albumin–Bilirubin (PALBI) grade	Various, mainly liver diseases
Prognostic Nutritional Index = Onodera’s Prognostic Nutritional Index	Various
SMART-COP	Pneumonia
Systemic Inflammation Score (SIS)	Malignancies

**Table 2:** Prognostic indices for specific patient groups, along with interpretations of the role of plasma albumin.

Index Name (Sorted Alphabetically)	Original Research Articles				Reviews/Systematic Reviews			
	Infl. ^1^	Nutr. ^2^	Misc. ^3^	None	Infl.	Nutr.	Misc.	None
Advanced Lung Cancer Inflammation Index (ALI)	[13,14,15,16,17]	[14,16,17,18,19,20]	[14]	[21,22]				
AIMS65				[23,24,25,26,27,28,29,30,31,32]			[33]	[34,35,36,37,38,39,40]
ALBI-TNM = ALBI-T Score		[41]	[41,42]	[43,44,45,46,47,48,49,50]				[51]
Albumin–Bilirubin (ALBI) Score			[52]	[53,54,55,56,57,58,59,60,61]	[62]	[63]	[62,64,65]	[66,67,68,69,70,71]
Child–Pugh Score		[72]	[72,73,74,75]	[74,76,77,78] [55,79,80]				[81,82,83,84]
International Prognostic score for advanced Hodgkin’s disease				[85,86,87,88,89,90,91,92,93,94]				
The International Society of Renal Nutrition and Metabolism (ISRNM) Protein Energy Wasting (PEW) Index	[95,96,97]	[95,96,97,98,99,100,101,102,103]		[104]	[105]	[105]	[105]	
International Staging System (ISS) for Multiple Myeloma	[106]			[107,108,109,110,111,112,113,114,115]	[116]		[116]	[117,118,119,120,121,122,123,124,125]
Malnutrition–Inflammation Score = Kalantar Score	[126,127]	[126,127,128,129,130,131,132,133,134]		[135]	[136,137,138,139,140]	[136,139,140]	[136,141]	
New England Spinal Metastasis Score	[142,143]	[142,143,144]		[145,146,147,148,149,150,151]				[152]
Nonalcoholic fatty liver disease (NAFLD) Fibrosis Score			[153]	[154,155,156,157,158,159,160,161,162]			[163,164]	[165,166,167,168,169,170,171,172]
SMART-COP				[173,174,175,176,177,178,179,180,181,182]				[183,184,185,186,187,188,189]
Total:	13	29	9	83	8	5	10	43

^1^ Inflammation. ^2^ Nutrition. ^3^ Miscellaneous.

**Table 3:** Prognostic indices for broader patient groups, along with interpretations of the role of plasma albumin.

Index Name (Sorted Alphabetically)	Non-Review Studies				Reviews/Systematic Reviews			
	Infl. ^1^	Nutr. ^2^	Misc. ^3^	None	Infl.	Nutr.	Misc.	None
Albumin/Alkaline Phosphate Ratio (AAPR)	[195,196,197,198,199,200,201]	[195,196,197,199,200,201,202,203,204]	[196,197,199,202,203,204]		[205]		[205]	
Albumin/Fibrinogen Ratio (AFR)	[206,207,208,209,210,211,212]	[206,207,210,211,212,213,214,215]	[212,214]		[216]	[216]		
Albumin/globulin ratio	[217,218,219,220,221,222]	[217,218,219,222,223,224]	[219]	[225,226]	[227,228,229,230,231,232]	[227,228,229,230,231,232,233,234]		
Acute Physiology, Age, Chronic Health Evaluation (APACHE) III Score				[235,236,237,238,239,240,241,242,243,244]				[245,246,247,248,249]
Blood Urea Nitrogen/Albumin Ratio	[250,251,252,253,254,255]	[250,251,252,253,254,255,256,257]	[251,252,253,255,256,257]	[258,259]	[260,261]			
C-reactive protein/Albumin ratio	[190,191,262,263,264,265,266,267]	[190,191,262,263,264,265,267,268]	[190,263,264,267]	[269]	[270,271,272,273,274,275]	[271,272,273,274,275,276,277,278,279]	[270,271,279]	
COntrolling NUTritional status (CONUT) Score	[192,193,280,281]	[193,194,280,281,282,283,284,285,286]	[286]		[287,288,289,290,291,292,293,294,295]	[287,288,289,290,291,292,293,294,295,296]	[288,289,290,294]	
Fibrinogen/Albumin ratio (FAR)	[297,298,299,300,301,302,303,304,305]	[298,299,300,301]	[300,306]		[307,308]	[307,308]		
Geriatric Nutritional Risk Index	[193,309,310,311]	[193,194,309,310,311,312,313,314,315]	[311]	[316]	[317,318]	[317,318,319,320,321,322,323,324,325,326]		
Glasgow Prognostic Score	[190,191,327,328,329]	[190,191,328,330]	[190]	[331,332,333,334]	[335,336,337,338,339,340,341,342]	[335,336,337,338,339,341,342]	[342]	[343,344]
Gustave Roussy Immune (GRIM) Score	[345]	[345,346,347]		[348,349,350,351,352,353,354]				
Hemoglobin, ALB, Lymphocyte, Platelet (HALP) Score	[355,356,357,358,359,360,361]	[355,356,358,359,360,362,363,364]			[365,366]	[365,366]	[365]	
Ischemia-modified Albumin/Albumin ratio			[367,368,369,370,371,372,373,374,375,376]					
Naples Prognostic Score	[377,378,379,380,381,382]	[377,378,379,380,381,382,383,384]	[382]	[385,386]				
Neutrophil (Percentage)/Albumin ratio	[387,388,389,390,391,392,393,394,395]	[387,391,392,394,396]	[387]					
Platelet–Albumin–Bilirubin (PALBI) grade	[397]	[398]	[398,399,400]	[43,401,402,403,404,405]		[406]	[406]	
Prognostic Nutritional Index = Onodera’s Prognostic Nutritional Index	[192,407,408,409,410]	[407,408,410,411,412,413,414]		[415]	[416,417,418,419,420,421]	[416,417,418,420,421,422,423,424,425]	[418,419]	
Systemic Inflammation Score (SIS)	[426,427,428,429,430,431,432,433]	[426,427,428,429,430,431,432,434,435]			[436]	[436]		
Total:	93	107	39	36	46	60	13	7

^1^ Inflammation. ^2^ Nutrition. ^3^ Miscellaneous.

**Table 4:** Prognostic indices for broader patient groups (distributed according to main disease entity).

Index Name (Sorted Alphabetically)	Main Disease Entity					
	Malignancy		Infection		Other	
	Original Research	Review	Original Research	Review	Original Research	Review
Albumin/Alkaline Phosphate Ratio (AAPR)	9		0		1	
Albumin/Fibrinogen Ratio (AFR)	7	2	0	0	3	0
Albumin/globulin ratio	7	7	0	1	3	0
APACHE III Score	0	0	3	0	7	5
Blood Urea Nitrogen/Albumin Ratio	1	0	5	2	4	0
C-reactive protein/Albumin ratio	3	9	3	1	4	0
COntrolling NUTritional status (CONUT) Score	4	7	0	0	6	3
Fibrinogen/Albumin ratio (FAR)	2	1	3	1	5	0
Geriatric Nutritional Risk Index	1 (1) ^1^	8	0	0	7 (1) ^1^	2
Glasgow Prognostic Score	7 (2) ^2^	10	0	0	1	0
Gustave Roussy Immune (GRIM) Score	10		0		0	
Hemoglobin, ALB, Lymphocyte, Platelet (HALP) Score	9	2	0	0	1	0
Ischemia-modified Albumin/Albumin ratio	0		1		9	
Naples Prognostic Score	9		0		1	
Neutrophil (Percentage)/Albumin ratio	0		2		8	
Platelet–Albumin–Bilirubin (PALBI) Grade	7	1	0	0	3	0
Prognostic Nutritional Index	5	8	2	0	2 (1) ^1^	2
Systemic Inflammation Score (SIS)	10	1	0	0	0	0
Total:	91 (3) ^3^	56	19	5	65 (2) ^3^	12

^1^ One additional study was also used for the COntrolling NUTritional status (CONUT) Score. ^2^ Two additional studies were also used for the C-reactive protein/Albumin ratio. ^3^ Studies occurring in two indices.

**Table 5:** Publications on indices according to authors’ continent and country.

Continent	Country	Related to Specific Disorders		Related to Broader Patient Groups	
		No. (%) of Studies	No. (%) of Reviews	No. (%) of Studies	No. (%) of Reviews
Unspecified	Two or more	15 (12.6)	14 (24.1)	8 (4.6)	4 (5.5)
Africa	Egypt	4 (3.4)			
	Nigeria		1 (1.7)		
America	Brazil	3 (2.5)	1 (1.7)		
	Canada	1 (0.8)	1 (1.7)		
	Mexico	2 (1.7)			
	Peru				2 (2.7)
	USA	11 (9.2)	12 (20.7)	4 (2.3)	3 (4.1)
Asia	China	29 (24.4)	7 (12.1)	72 (41.1)	52 (71.2)
	India	2 (1.7)		2 (1.1)	
	Iran		1 (1.7)	1 (0.6)	
	Japan	15 (12.6)	3 (5.2)	40 (22.9)	1 (1.4)
	Korea	5 (4.2)	1 (1.7)	3 (1.7)	1 (1.4)
	Malaysia	1 (0.8)			
	Saudi Arabia	1 (0.8)		1 (0.6)	
	Singapore	1 (0.8)	1 (1.7)		
	Taiwan	5 (4.2)		2 (1.1)	6 (8.2)
	Thailand	2 (1.7)			
	Turkey	6 (5.0)		27 (15.4)	
	United Arab Emirates	1 (0.8)			
Australia	Australia			3 (1.7)	
	New Zealand	1 (0.8)		1 (0.6)	
Europe	Croatia	1 (0.8)		1 (0.6)	
	Denmark			2 (1.1)	
	France	3 (2.5)	2 (3.4)	1 (0.6)	
	Germany		1 (1.7)	1 (0.6)	
	Greece				1 (1.4)
	Italy	5 (4.2)	4 (6.9)	4 (2.3)	1 (1.4)
	Netherlands	1 (0.8)	1 (1.7)		
	Poland				1 (1.4)
	Portugal	1 (0.8)			
	Romania	1 (0.8)		1 (0.6)	
	Spain	1 (0.8)	1 (1.7)		
	Sweden	1 (0.8)			
	Switzerland		1 (1.7)		
	United Kingdom		6 (10.3)	1 (0.6)	1 (1.4)
	Total:	119	58	175	73

## Appendix A

**Table A1:** Indices (sorted alphabetically), search strings, and numbers of hits (retrieved from PubMed, 18 February 2022).

Index Name	Search String	No. of Hits	
		PubMed ^1^	Manual ^2^
Advanced Lung Cancer Inflammation Index (ALI)	“advanced lung cancer inflammation index”	54	≥10
Age, Comorbidities, and albumin (ACA) index	“ACA index”	4	2
AGR-PLR Score (APS)	“AGR-PLR index”	2	2
AIMS65	“AIMS65”	105	≥10
ALBI-TNM = ALBI-T Score	“ALBI-T” OR “ALBI-T score”	23	≥10
Albumin–Bilirubin (ALBI) Score	“albumin-bilirubin score” OR “albumin-bilirubin grade” OR “albumin-bilirubin index” OR “ALBI”	1282	≥10
Albumin–Globulin score (AGS)	“albumin-globulin score”	7	7
Albumin–Indocyanine Green Evaluation (ALICE) Grade	“albumin-indocyanine green evaluation grade” OR “ALICE grade”	7	6
Albumin/Alkaline Phosphate Ratio (AAPR)	“albumin to alkaline phosphate ratio” OR “AAPR”	181	≥10
Albumin/Fibrinogen Ratio (AFR)	“albumin to fibrinogen ratio”	34	≥10
Albumin/Gamma-Glutamyltransferase (AGR) Ratio	“albumin to gamma-glutamyltransferase ratio” OR “AGR ratio”	4	2
Albumin/globulin ratio	“albumin to globulin ratio”	208	≥10
Acute Physiology, Age, Chronic Health Evaluation (APACHE) III Score	“APACHE III” OR “APACHE 3”	667	≥10
BALAD Score	“BALAD score”	5	5
BALAD-2 Score	“BALAD-2”	9	9
Blood Urea Nitrogen/Albumin Ratio	“blood urea nitrogen to albumin ratio” OR “BUN to albumin ratio”	298	≥10
C-reactive protein/albumin ratio	“C-reactive protein to albumin ratio” OR “CRP to albumin ratio”	240	≥10
Child–Pugh Score	“child-pugh score”	2214	≥10
Cirrhosis acute gastrointestinal bleeding (CAGIB) score	“CAGIB score”	2	2
COntrolling NUTritional status (CONUT) Score	“controlling nutritional status score” OR “CONUT score”	363	≥10
Creatinine/albumin ratio	“serum creatinine” AND “albumin” AND “ratio”	1365	1
DCTA scoring system	“DCTA scoring system”	4	1
Expanded A-drop score	“expanded A-drop score”	11	1
EZ-ALBI score	“EZ-ALBI score”	2	2
Fibrinogen/albumin ratio (FAR)	“fibrinogen to albumin ratio”	63	≥10
Gamma-Glutamyltransferase/albumin ratio	“gamma-glutamyltransferase to albumin ratio”	1	1
Geriatric Nutritional Risk Index	“geriatric nutritional risk index”	481	≥10
Glasgow multifactor prognostic scoring systems	“glasgow multifactor prognostic scoring system”	2	2
Glasgow Prognostic Score	“glasgow prognostic score”	932	≥10
Gustave Roussy Immune (GRIM) Score	“gustave roussy immune score” OR “GRIM score”	12	≥10
Hemoglobin albumin Lymphocytes Neutrophils (HLAN) Score	“hemoglobin albumin lymphocytes neutrophils score” OR “HLAN score”	66	1
Hemoglobin, albumin, Lymphocyte, Platelet (HALP) Score	“hemoglobin albumin lymphocyte platelet score” OR “HALP score”	61	≥10
Inflammatory Prognostic Index (IPI)	“inflammatory prognostic index”	5	4
The Instrumental Activities of Daily Living (IADL) Scale and the ACA Index	“instrumental activities of daily living scale and the ACA index” OR (“IADL” AND “ACA index”)	1	1
International Normalized Ratio/albumin Ratio (PTAR)	“international normalized ratio to albumin Ratio” OR “PTAR”	36	4
International Prognostic score for advanced Hodgkin’s disease	“international prognostic score” AND “Hodgkin”	163	≥10
The International Society of Renal Nutrition and Metabolism (ISRNM) protein energy wasting (PEW) index	“international society of renal nutrition and metabolism protein energy wasting index” OR (“ISRNM” AND “PEW”)	32	≥10
International Staging System (ISS) for multiple myeloma	“international staging system” AND “myeloma”	621	≥10
Ischemia-modified albumin/albumin ratio	“ischemia modified albumin to albumin ratio”	103	≥10
Lactate dehydrogenase/albumin ratio	“lactate dehydrogenase to albumin ratio” OR “LDH to albumin ratio”	9	9
Lactate/albumin ratio	“lactate to albumin ratio”	3	3
LANR	“LANR”	24	1
Malnutrition–Inflammation Score = Kalantar Score	“malnutrition-inflammation score” OR “kalantar score”	235	≥10
Memorial Sloan Kettering Prognostic Score (MPS)	“memorial sloan kettering prognostic score”	2	2
Nonalcoholic fatty liver disease (NAFLD) Fibrosis Score	“NAFLD fibrosis score”	440	≥10
Naples Prognostic Score	“naples prognostic score”	21	≥10
Neutrophil Percentage/albumin Ratio (NPAR) ^3^	“neutrophil percentage to albumin ratio”	12	≥10
Neutrophil/albumin ratio ^3^	“neutrophil to albumin ratio”	12	≥10
New England Spinal Metastasis Score	“new england spinal metastasis score”	14	≥10
NOBLADS	“NOBLADS”	8	8
Nutritional and inflammatory status (NIS) ratio	“NIS ratio”	3	1
Platelet–albumin–Bilirubin (PALBI) grade	”platelet albumin bilirubin grade” OR ”PALBI grade”	21	≥10
Prognostic Inflammation Score (PIS)	“prognostic inflammation score”	3	2
Prognostic Inflammatory Nutritional Index (PINI)	“prognostic inflammatory nutritional index”	2	1
Prognostic Nutritional Index = Onodera’s Prognostic Nutritional Index	“prognostic nutritional index”	1141	≥10
Red cell distribution width/albumin ratio	“red cell distribution width albumin ratio” OR “RDW albumin ratio”	242	8
SMART-COP	“SMART-COP”	48	≥10
Systemic Inflammation Score (SIS)	“systemic inflammation score”	52	≥10
SWOG Stage	“SWOG” AND albumin	12	4

^1^ Number of hits retrieved in PubMed (https://pubmed.ncbi.nlm.nih.gov/?otool=idkvouhlib, accessed on 18 February 2022). ^2^ Number of English-language publications (excluding casuistic studies) (0–9, ≥10) found relevant for the index by manually looking through the results retrieved via PubMed (sorted by and retrieved from the newest publications). ^3^ Through analyzing the studies and reviews, it was revealed that the terms “Neutrophil Percentage to albumin Ratio” and “Neutrophil to albumin Ratio” were used interchangeably; therefore, the two terms have been merged in this article.

**Table A2:** Indices that incorporate serum or plasma albumin and their computation (index name sorted alphabetically). All biochemistry parameters refer to levels in blood, serum, or plasma.

Index Name	Computation	References
Advanced Lung Cancer Inflammation Index (ALI)	[BMI (kg/m^2^) × ALB (g/dL)]/NLR	[461]
Age, Comorbidities, and ALB (ACA) index	1 point for each of: Age > 75 years; ALB < 3.7 g/dL; Charlson Comorbidity Index Score ≥ 3	[462,463]
AGR-PLR Score (APS)	AGR = ALB (g/L)/globulins (g/L) PLR = Platelet/Lymphocyte Ratio APS score 0: PLR ≤ 132 and AGR > 1.75; score 1: PLR > 132 or AGR ≤ 1.75; score 2: PLR > 132 and AGR ≤ 1.75	[464]
AIMS65	1 point for each of: ALB < 3.0 mg/dL; INR > 1.5; Altered mental status; Systolic blood pressure < 90 mm Hg; Age > 65 years	[465]
ALBI-TNM = ALBI-T Score	ALBI grade + TNM stage of LCSGJ – 2 ALBI grade: See “ALB-Bilirubin (ALBI) Score” TNM stage of Liver Cancer Study Group of Japan (LCSGJ): Stage I: T1N0M0; Stage II: T2N0M0; Stage III: T3N0M0; Stage IVa: T4N0M0, or any TN1M0; Stage IVb: Any TN0-N1M1	[50]
ALB-Bilirubin (ALBI) Score	(log10 [bilirubin (μmol/L)] × 0.66) + (ALB (g/L) × [−0.085])	[466]
ALB-Globulin score (AGS) ^1^	Score 0: ALB > 35 g/L and globulin ≤ 35 g/L; Score 1: ALB ≤ 35 g/L or globulin > 35 g/L; Score 2: ALB ≤ 35 g/L and globulin > 35 g/L	[467]
ALB-Indocyanine Green Evaluation (ALICE) Grade	0.663 × log10 [ICG R15 (%)] − 0.0718 × ALB (g/L) ICG R15 (%) = retention rate of Indocyanine Green, 15 min after its infusion	[468,469]
ALB/Alkaline Phosphate Ratio (AAPR)	ALB (g/L)/Alkaline phosphatase (IU/L)	[201]
ALB/Fibrinogen Ratio (AFR)	ALB (g/L)/Fibrinogen (g/L)	[470]
ALB/Gamma-Glutamyltransferase (AGR) Ratio	ALB (g/L)/Gamma-Glutamyltransferase (IU/L)	[471]
ALB/globulin ratio	ALB (g/dL)/[Total protein (g/dL) − ALB (g/dL)]	[472]
Acute Physiology, Age, Chronic Health Evaluation (APACHE) III Score	ALB: 11 points: ≤19 g/L; 6 points: 20–24 g/L; 0 points: 25–44 g/L; 4 points: ≥45 g/L + numerous other parameters (APACHE III Score, range 0–299)	[2]
BALAD Score	Bilirubin (mg/dL)/ALB (g/dL) score: 0 points: <1.0/>3.5; 1 point: 1.0–2.0/2.8–3.5; 2 points: >2.0/<2.8 Scores for elevated tumor markers, 1 point for each of the: α-Fetoprotein > 400 ng/mL; α-Fetoprotein-L3 > 15% (of α-Fetoprotein); Des-ϒ-Carboxyprothrombin > 100 mAU/mL BALAD Score: Bilirubin/ALB score + scores for elevated tumor markers	[473]
BALAD-2 Score	BALAD-2 Score = 0.02 × (α-Fetoprotein − 2.57) + 0.012 × (α-Fetoprotein-L3 − 14.19) + 0.19 × (ln [Des-ϒ-Carboxyprothrombin] − 1.93) + 0.17 × (Bilirubin^1/2^ − 4.50) − 0.09 × (ALB − 35.11) Units: α-Fetoprotein (1000× ng/mL); α-Fetoprotein-L3 (% of α–Fetoprotein); Des-ϒ-Carboxyprothrombin (1000× ng/mL); Bilirubin (μmol/L); ALB (g/L)	[474,475]
Blood Urea Nitrogen/ALB Ratio	Blood urea nitrogen (mg/dL)/ALB (g/dL)	[251]
C-reactive protein/ALB ratio	C-reactive protein (mg/L)/ALB (g/L)	[476]
Child–Pugh Score	1, 2, 3 points, respectively, for each of: Total bilirubin: <2, 2–3, >3 mg/dL; ALB: >3.5, 2.8–3.5, <2.8 g/dL; INR: <1.7, 1.7–2.2, >2.2; Ascites: Absent, Slight, Moderate; Encephalopathy: None, Grade 1–2, Grade 3–4	[477]
Cirrhosis acute gastrointestinal bleeding (CAGIB) score	Diabetes (yes = 1, no = 0) × 1.040 + Hepatocellular carcinoma (yes = 1, no = 0) × 0.974 + Bilirubin (μmol/L) × 0.005 − ALB (g/L) × 0.091 + Alanine aminotransferase (IU/L) × 0.001 + Serum creatinine (μmol/L) × 0.012 − 3.964	[478]
COntrolling NUTritional status (CONUT) Score	ALB, g/dL (points): 3.5–4.5 (0), 3.0–3.49 (2), 2.5–2.9 (4), <2.5 (6) Lymphocytes/mL (points): >1600 (0), 1200–1599 (1), 800–1199 (2), <800 (3) Cholesterol, mg/dL (points): >180 (0), 140–180 (1), 100–139 (2), <100 (3)	[479]
Creatinine/ALB ratio	Creatinine (μmol/L)/ALB (g/L)	[480]
DCTA scoring system	1 point for each of: D-dimer > 0.63 mg/L; Cholesterol ≤ 3.68 mmol/L; ALB ≤ 34 g/L; High sensitivity cardiac troponin T > 24.06 pg/mL	[481]
Expanded A-drop score	1 point for each of: Age ≥ 70 years (male) or ≥75 years (female); Blood urea nitrogen ≥ 21 mg/dL or dehydration; Oxygen tension ≤ 90% (PaO_2_ ≤ 60 mmHg); Confusion; Systolic blood pressure ≤ 90 mmHg; Malignancy; Heart rate ≥ 100/min; ALB ≤ 3.09 g/dL; Lactate > 1.7 mmol/L; N-terminal pro-brain natriuretic peptide > 500 pg/mL	[482]
EZ-ALBI score	Total bilirubin (mg/dL) − 9 × ALB (g/dL)	[483]
Fibrinogen/ALB ratio (FAR)	Fibrinogen (mg/dL)/ALB (g/dL)	[484]
Gamma-Glutamyltransferase/ALB ratio	Gamma-Glutamyltransferase (IU/L)/ALB (g/L)	[485]
Geriatric Nutritional Risk Index	(1.489 × ALB [g/dL]) + (41.7 × [body weight/ideal body weight]) Lorentz-formula, for calculating the ideal body-weight (w) of a subject: For men: w = (height [cm] − 100) − ((height − 150)/4) For women: w = (height − 100) − ((height − 150)/2.5)	[486]
Glasgow multifactor prognostic scoring systems	In all three systems [487,488,489], 1 point for each of: Neutrophils (>15 × 10^9^/L); Blood glucose (>10 mmol/L); Serum urea (>16 mmol/L); Arterial oxygen saturation (<8 kPa); Serum calcium (<2 mmol/L); ALB (<32 g/L); Serum lactate dehydrogenase (>600 units/L) In [487,489]: 1 point for age > 55 years In [487] and [488]: 1 point for serum transaminase >100 and >200 units/L, respectively	[487,488,489,490]
Glasgow Prognostic Score ^2^	0 points: C-Reactive protein (CRP) ≥ 10 mg/L and ALB ≥ 35g/L 1 point: CRP > 10 mg/L or ALB < 35 g/L 2 points: CRP > 10 mg/L and ALB < 35 g/L	[491,492]
Gustave Roussy Immune (GRIM) Score	1 point for each of: Lactate dehydrogenase > upper limit normal; ALB < 35 g/L; NLR > 6	[354]
Hemoglobin ALB Lymphocytes Neutrophils (HLAN) Score	(Hemoglobin [g/L] × Lymphocytes [/L] × ALB [g/L])/Neutrophils (/L)/100	[493]
Hemoglobin, ALB, Lymphocyte, Platelet (HALP) Score	(Hemoglobin [g/L] × ALB [g/L] × Lymphocyte [/L])/Thrombocytes (/L).	[364]
Inflammatory Prognostic Index (IPI)	(C-reactive protein (mg/L) × NLR)/ALB (g/L)	[494]
The Instrumental Activities of Daily Living (IADL) Scale and the ACA Index	IADL scores of 6 to 7 and ACA score 1: 1 point IADL scores ≤ 5 and ACA scores 2 or 3: 2 points IADL ACA (IACA) Index: Score 0 = low risk; Score 1–2 = intermediate risk; Score 3–4 = high risk ACA Score: See “Age, Comorbidities, and ALB (ACA) index” in this table	[495,496]
International Normalized Ratio/ALB Ratio (PTAR)	INR/ALB (g/dL)	[497]
International Prognostic score for advanced Hodgkin’s disease	1 point for each of: ALB < 4 g/dL; Hemoglobin < 10.5 g/dL; Male sex; Stage IV disease; Age ≥ 45 years; Neutrophils ≥ 15,000/mm^3^; Lymphocytes < 600/mm^3^, or <8% of neutrophil count	[498]
The International Society of Renal Nutrition and Metabolism (ISRNM) protein energy wasting (PEW) index	1 point for each of: ALB < 38 g/L; BMI ≤ 23 kg/m^2^; Serum creatinine ≤ 818 μmol/L; Protein intake assessed by the normalized protein catabolic rate ≤ 0.8 g/kg/day	[499]
International Staging System (ISS) for multiple myeloma	Stage I: Serum beta2-microglobulin < 3.5 mg/L and ALB ≥ 3.5 g/dL; Stage II: neither stage I nor III; Stage III: Serum beta2-microglobulin ≥ 5.5 mg/L. Two categories for stage II: Serum beta2-microglobulin < 3.5 mg/L/ALB < 3.5 g/dL; Serum beta2-microglobulin 3.5 to <5.5 mg/L, irrespective of the ALB level	[500]
Ischemia-modified ALB/ ALB ratio	Ischemia-modified ALB (Absorbance units [ABSU])/ALB (g/dL)	[376]
Lactate dehydrogenase/ALB ratio	Lactate dehydrogenase (IU/L)/ALB (g/dL)	[501]
Lactate/ALB ratio	Lactate (mmol/L)/ALB (g/dL)	[502]
LANR	[Lymphocytes (10^9^/L) × ALB (g/L)]/neutrophils (10^9^/L)	[503]
Malnutrition–Inflammation Score = Kalantar Score	Three factors were amended to the Subjective Global Assessment (SGA), with its seven factors (each of 0–3 points), thus rendering 0–30 points for the Malnutrition–Inflammation Score: BMI (kg/m^2^): 0 points: ≥20; 1 point: 18–19.99; 2 points: 16–17.99; 3 points: <16 ALB (g/dL): 0 points: ≥4.0; 1 point: 3.5–3.9; 2 points: 3.0–3.4; 3 points: <3.0 Total iron binding capacity (mg/dL): 0 points: <250; 1 point: 200–249; 2 points: 150–199; 3 points: <150 (Suggested equivalent increments for serum transferrin are >200, 170–199, 140–169, <140 mg/dL, respectively)	[504]
Memorial Sloan Kettering Prognostic Score (MPS)	0 points: ALB ≥ 4 g/dL and NLR ≤4; 1 point: ALB < 4 g/dL or NLR >4; 2 points: ALB <4 g/dL and NLR >4	[505]
Nonalcoholic fatty liver disease (NAFLD) Fibrosis Score	−1.675 + 0.037 × Age (years) + 0.094 × BMI (kg/m^2^) + 1.13 × (impaired fasting glucose)/diabetes (yes = 1, no = 0) + 0.99 × Aspartattransaminase/Alaninaminotransferase ratio − 0.013 × Thrombocytes (10^9^/L) − 0.66 × ALB (g/dL)	[506]
Naples Prognostic Score	1 point for each of: ALB < 4 g/dL; Cholesterol ≤ 180 mg/dL; NLR > 2.96; Lymphocyte/Monocyte Ratio ≤ 4.44	[507]
Neutrophil (Percentage)/ALB Ratio (NPAR) ^3^	Neutrophil percentage (X% recorded as X)/ALB (g/L) Or Neutrophils (10^9^/L)/ALB (g/dL)	[508,509]
New England Spinal Metastasis Score	Modified Bauer Score = 0 or 1 point if ≤2 or ≥3, respectively, of the following criteria: no visceral metastases; primary tumor is not lung cancer; primary tumor is breast, renal, lymphoma, or myeloma; single skeleton metastasis 1 point for each of: independent ambulatory function; ALB ≥ 3.5 g/dL	[150,510]
NOBLADS	1 point for each of: Non-steroid anti-inflammatory drugs; no diarrhea; no abdominal tenderness; systolic blood pressure ≤ 100 mm Hg; antiplatelet drugs (non-aspirin); ALB < 3.0 g/dL; Charlson Comorbidity Index Score ≥ 2; syncope	[463,511]
Nutritional and inflammatory status (NIS) ratio	(C-reactive protein [mg/L] × alpha-1 acid glycoprotein [mg/L])/(ALB [g/L] × pre-ALB [mg/L])	[512]
Platelet-ALB-Bilirubin (PALBI) grade	2.02 × log10(bilirubin) (μmol/L) − 0.37 × [log10(bilirubin)]^2^ − 0.04 × ALB (g/L) − 3.48 × log10(Thrombocytes) + 1.01 × log10(Thrombocytes)	[399]
Prognostic Inflammation Score (PIS) ^4^	0 point: ALB: >40 g/L and NLR ≤ 3.43; 1 point: ALB: ≤40 g/L or NLR > 3.43; 2 points: ALB: ≤40 g/L and NLR > 3.43	[513]
Prognostic Inflammatory Nutritional Index (PINI)	(alpha-1-acid glycoprotein [mg/L] + C-reactive protein [mg/L])/(ALB [g/dL] + pre-ALB [mg/L])	[514]
Prognostic Nutritional Index = Onodera’s Prognostic Nutritional Index ^5^	ALB (g/L) + 5 × Lymphocytes (10^9^/L)	[515,516]
Red cell distribution width/ALB ratio	Red cell distribution width (%)/ALB (g/dL)	[517]
SMART-COP (systolic blood pressure, multilobar chest radiography involvement, albumin level, respiratory rate, tachycardia, confusion, oxygenation, and arterial pH)	1 point for each of: Multiple lung lobes involvement on chest X-ray; ALB < 3.5 g/dL; Respiratory rate > 30 N/min; heart rate > 125 beats/min; confusion (acute) 2 points for each of: Systolic blood pressure < 90 mmHg; Oxygen saturation < 90%); potential pH < 7.35	[518]
Systemic Inflammation Score (SIS) ^6^	Score 0: ALB ≥ 4.0 g/dL and Lymphocyte/Monocyte Ratio ≥ 4.44; Score 1: ALB < 4.0 g/dL or Lymphocyte/Monocyte Ratio < 4.44; Score 2: ALB < 4.0 g/dL and Lymphocyte/Monocyte Ratio < 4.44	[519]
SWOG Stage	Stage1: Serum ß2 microglobulin < 2.5 mg/L; Stage 2: 2.5 ≤ Serum ß2 microglobulin < 5.5 mg/L; Stage 3: Serum ß2 microglobulin ≥ 5.5 mg/L and ALB ≥ 30 g/L; Stage 4: Serum ß2 microglobulin ≥ 5.5 mg/L and ALB <30 g/L	[520]

ALB = albumin; BMI = body mass index; INR = International normalized Ratio; NLR = neutrophil to lymphocyte ratio. ^1^ Other cut-off levels are also reported for AGS [521]. ^2^ In the Modified Glasgow Prognostic Score, 1 point for ALB < 35 g/L and C-reactive protein < 10 mg/L is omitted [491]. ^3^ The terms ”Neutrohil to ALB ratio” and “Neutrophil percentage to ALB ratio” are used interchangeably, and the same applies to the way they are computed (either as a number or as a percentage of neutrophils). Consequently, the two terms have been merged in this article. ^4^ There is also a score designated to the Prognostic Inflammation Score (PIS), which does not incorporate ALB [522]. ^5^ The Prognostic nutritional index (PNI), initially reported by [523], was previously calculated differently, but Onodera et al. [516] developed the present index in 1984 [422]. ^6^ Other cut-off levels are reported; e.g., for the Modified Systemic Inflammation score (mSIS) [524] or the Adapted Systemic Inflammation Score (aSIS) [435].
